# Cardiac Magnetic Resonance—Detected Acute Myocardial Edema as Predictor of Favourable Prognosis: A Comprehensive Review

**DOI:** 10.3390/jcdd10080319

**Published:** 2023-07-27

**Authors:** Giulio Sinigiani, Laura De Michieli, Giorgio De Conti, Fabrizio Ricci, Manuel De Lazzari, Federico Migliore, Martina Perazzolo Marra, Alessandro Zorzi, Domenico Corrado, Alberto Cipriani

**Affiliations:** 1 Department of Cardiac, Thoracic, Vascular Sciences and Public Health, University of Padua, 35128 Padua, Italy; 2 Radiology Unit, Department of Medicine, Institute of Radiology, University of Padua, 35128 Padua, Italy; 3 Department of Neuroscience, Imaging and Clinical Sciences, G. D’Annunzio University of Chieti-Pescara, 66100 Chieti, Italy

**Keywords:** myocardial edema, cardiac magnetic resonance, prognosis

## Abstract

Acute myocardial edema (AME) is increased water content in the myocardium and represents the first and transient pathophysiological response to an acute myocardial injury. In-vivo and non-invasive evaluation is feasible with cardiac magnetic resonance (CMR), which is a powerful imaging technique capable of tissue characterization. In the clinical setting, early demonstration of AME has a recognized diagnostic value for acute coronary syndromes and acute myocarditis, although its prognostic value is not well established. This article provides a comprehensive narrative review on the clinical meaning of AME in heart diseases. In particular, the available evidence of a possible favourable prognostic value in several clinical scenarios is addressed.

## 1. Introduction

Acute myocardial edema (AME) is increased water content in the myocardium and represents the first pathophysiological response to an acute myocardial injury [1,2]. Physiologically, myocardial water is distributed in the intracellular (77%) and in the intravascular compartment (23%). The presence of Na^+^/K^+^ATP-dependent pumps and the oncotic pressure carried out by intramyocellular protein counteract diffusion, making the interstitial amount of water virtual [3]. The increase of myocardial water in AME is first an accumulation of intracellular water (cytogenic edema), followed by build-up of interstitial water (vasogenic edema). The cause of acute myocardial injury characterizes the mechanism responsible for AME. For example, in ischemia, an acidosis-related reduction in the activity of membrane pumps is primarily involved, whereas in myocardial inflammation, vasodilatation and increased capillary permeability are the most important factors [4]. 

AME is a transient phenomenon, resolving quickly in absence of necrosis and/or fibrosis. Accordingly, it may predict reversibility and healing of the myocardial injury [1]. The in vivo and non-invasive evaluation of AME is feasible with cardiac magnetic resonance (CMR), which is a powerful tool to assess myocardial tissue characterization by means of multiple techniques such as T1-, T2-weighted and post-contrast sequences [5,6]. In detail, an increased signal intensity on T2-weighted sequences is suggestive of edema, whereas an increased intensity on T1-weighted images in combination with the presence of late gadolinium enhancement (LGE) on post-contrast sequences is suggestive of necrosis and/or fibrosis. Upon these principles, myocardial T1 and T2 mapping techniques allow an even more accurate and sensitive characterization of the myocardium [7,8]. The combination of different CMR sequences provides the unique possibility to distinguish an acute and potentially reversible injury from a chronic and irreversible myocardial lesion in a wide spectrum of heart diseases. In the clinical setting, an early demonstration of AME has a recognized diagnostic value in some acute diseases, such as coronary syndromes or acute myocarditis [9]. Conversely, its prognostic value is not well established, even if current perspectives consider AME detected by CMR as a possible predictor of favourable prognosis.

This article provides a comprehensive narrative review on the clinical meaning of AME in heart diseases. In particular, the available evidence of a possible favourable prognostic value in different clinical scenarios, including acute coronary syndromes, acute myocarditis, Tako-Tsubo syndrome (TTS) and resuscitated sudden cardiac death, will be addressed. 

## 2. Acute Coronary Syndromes

AME, in the context of acute coronary syndromes, is seen in almost all patients. In line with the ischemic wave-front phenomenon, it spreads from the subendocardium to the whole wall thickness of the myocardium supplied by the occluded artery. The possibility of its detection with T2 sequences is crucial for diagnostic and prognostic assessment. In particular, for this latter, the hyperintense myocardium in such sequences defines the so-called “area at risk” [10,11]. This is the myocardium supplied by occluded artery but still viable, unless a prolonged and sustained ischemia occurs. Conversely, post contrast T1 sequences could reveal the effective infarcted area, the so-called infarct size [10]. The difference between these two regions is the salvaged myocardium. Its assessment by this method is reliable and validated against perfusion scintigraphy and coronary angiogram [2]. As demonstrated by Eitel and colleagues, the greater the salvaged myocardium, the better the prognosis, with a ten-fold reduction in prevalence of major adverse cardiovascular events at 6 months [12]. A further study by Li and colleagues confirmed this finding in a longer follow up of 8 months [13]. Given these prognostic implications, the temporal evolution of AME is worth considering for the definition of the best timing of the evaluation of salvaged myocardium. As shown by Dall’Armellina and colleagues, edema seems to be stable in the first week after ischemia [14]. A later study by Fernandez–Jimenz and colleagues revealed a possible bimodal increase, with a first peak in 24-h and a second peak at day 7 after ischemia [15]. The first wave is hypothesized to be related to a vasogenic edema after reperfusion, whereas the second one to myocardial healing [16]. Nevertheless, recent studies [17,18] have questioned this bimodal behaviour, suggesting that other factors, including microvascular obstruction and intramyocardial haemorrhage, could have a role in this phenomenon. Taken together, these studies point to the importance of timing in the assessment of myocardial edema in acute myocardial infarction, a topic still debated in literature [10,19]. Despite this limitation, an imaging scan performed within two weeks from the event is considered acceptable [20]. In summary, the visualization of extensive area at risk, either ischemic or post-reperfusion, in presence of small myocardial infarction size, is an index of a good amount of salvaged myocardium, which is a contributor to a favourable prognosis and left ventricle remodelling [21]. 

## 3. Acute Myocarditis

**Figure 1:**
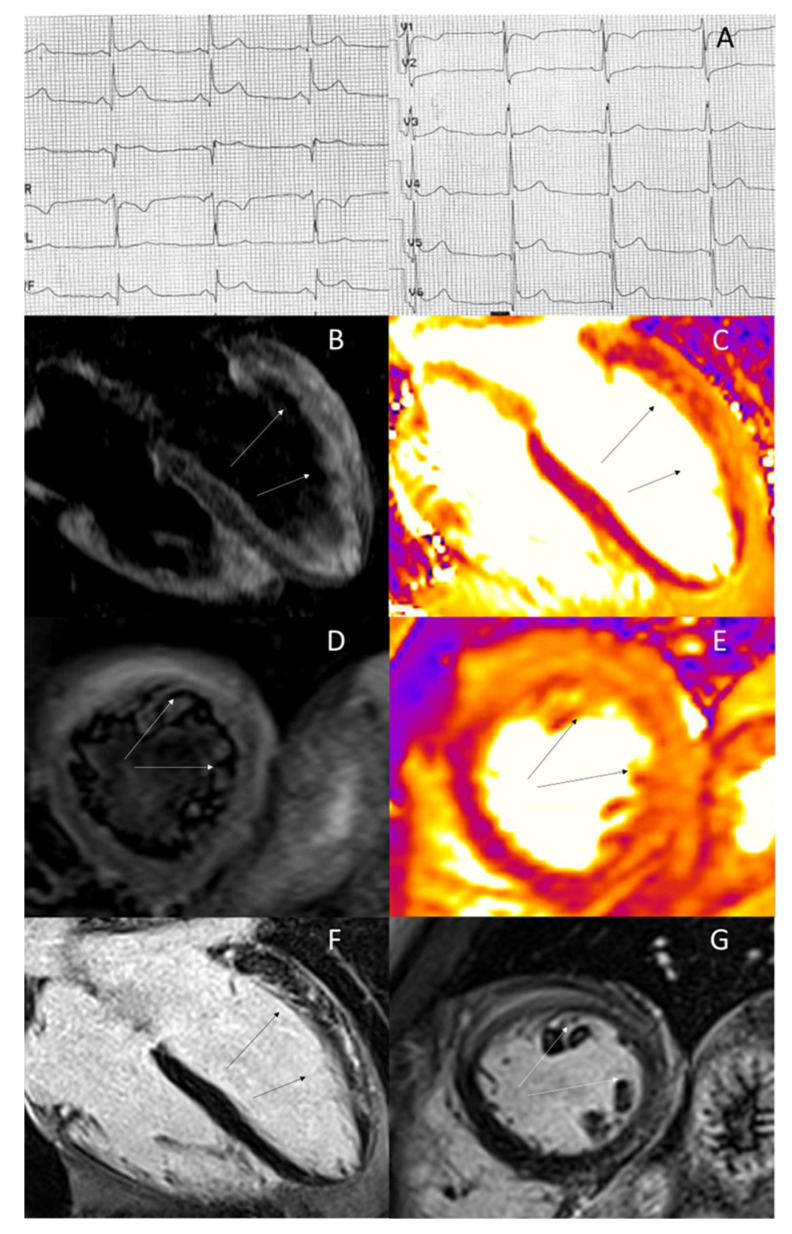
35-year-old man admitted with chest pain. ECG showing ST tract elevation in inferior and V5–V6 leads (**A**). Cardiac magnetic resonance (CMR) T2-weighted (**B**,**D**) and T2-mapping (**C**,**E**) images showing myocardial edema in the basal and mid segments of the anterior and antero-lateral wall. CMR late gadolinium enhancement (LGE) sequences (**F**,**G**) showing subepicardial–mid wall LGE in the same regions (arrows), suggesting acute myocarditis.

The detection of AME is a cornerstone of non-invasive diagnosis of clinically suspected acute myocarditis [5,22,23]. Its prevalence ranges from 60% to over 90%. Typically, it has a mid-wall or sub-epicardial distribution pattern, visible throughout the whole myocardium, but most often in basal and mid-ventricular septal or infero-lateral segment of the left ventricle [24] (Figure 1). From a prognostic perspective, its presence seems to be associated with favourable outcome [25]. In a recent retrospective study including 388 patients with clinically suspected myocarditis, the absence of edema was associated with adverse outcome [26]. This finding is in line with the evidence of recovery in left ventricular ejection fraction in patients with myocarditis and greater global myocardial inflammation [27]. Furthermore, in a study by Aquaro et al. of 187 patients with suspected myocarditis, in those with evidence of edema in the baseline scan, a complete recovery was observed in the follow up CMR after 6 months. The possible presence of edema did not show adverse prognostic significance over 7 years of follow-up [28]. The favourable prognostic role of AME in clinically suspected or biopsy-proven myocarditis was further confirmed despite its extension [29,30]. All these findings reinforce the idea of AME, but not LGE, as a marker of reversible injury and good prognosis [1,30,31,32,33]. Anyway, LGE in a context of myocarditis is not a definitive sign of irreversible myocardial damage. Inflammatory cells infiltration and AME itself could lead to an increased interstitial space and to a slowed wash-out of the gadolinium-based contrast media [28]. Consequently, in a large proportion of cases, the LGE is reduced or absent at a 6-month follow up CMR [34], and this is an adjunctive marker of favourable prognosis [28]. This evidence confirms that the role of CMR is not limited to the diagnosis of this disease and reinforces its value in the management of affected patients. 

## 4. Takotsubo Syndrome

**Figure 2:**
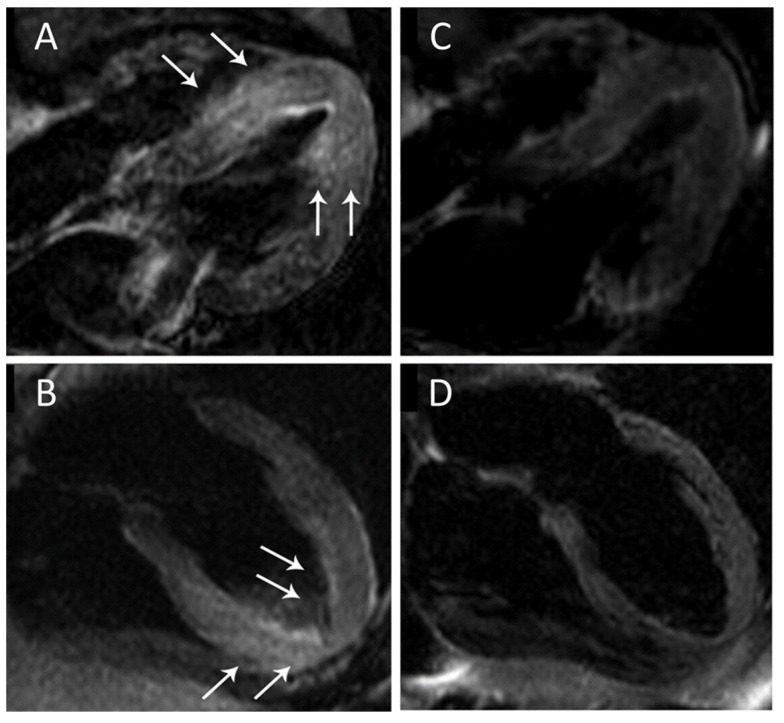
T2-weighted sequences showing myocardial edema during acute phase (**left**) and follow-up (**right**). Four-chamber view showing edema in the left ventricular mid and apical inferoseptal and anterolateral segments (arrows in (**A**,**B**)). T2-weighted sequences on follow-up scan showing disappearance of edema (**C**,**D**). Adapted with kind permission from Migliore et al. [42].

AME is a relevant finding in the diagnosis of TTS, helping the differential diagnosis [35] with both acute coronary syndromes and acute myocarditis [36]. Usually, it shows a transmural extension, in both ballooning (Figure 2) and non–ballooning segments [37], and in the right ventricle [38]. As reported by Eitel and colleagues in a study with 199 patients, up to 80% of them had AME, depending upon the time between CMR evaluation and acute presentation [39]. In the same cohort, a complete resolution of edema and left ventricular dysfunction was observed in almost all patients. Furthermore, a prospective study by Perazzolo Marra et al., demonstrated complete regression of myocardial edema and functional recovery without evidence of fibrosis in the follow up CMR at 3 months [40]. These findings confirm that the presence of AME without LGE has favourable prognostic implications [41]. The detection of AME is useful also for the clinical management of TTS patients. Indeed, CMR and ECG studies demonstrated the association between AME and T-wave inversion or prolongation of QT interval. Migliore and colleagues [42] reported some cases of patients with a variety of disease including TTS syndrome showing both Wellens’ ECG pattern with prolongation of QT corrected (QTc) interval and LV systolic dysfunction in association with edema on CMR. After 6–8 weeks of follow-up, ECG abnormalities and systolic dysfunction resolved, along with the resolution of myocardial edema. Also, Zorzi and colleagues [43] reported the case of a patient with atypical “midventricular” TTS presenting with T-wave inversion and isolated QTc interval prolongation in the lateral leads. A subsequent CMR demonstrated myocardial edema in the mid-lateral wall, in line with the ECG abnormalities. The mentioned ECG changes account for most of the life-threatening arrhythmias described in the syndrome, occurring in the subacute phase of the disease [44]. Indeed, the majority of them are related to QTc prolongation, with pause-dependent torsades de pointes degenerating into ventricular fibrillation (VF), even if also long-short sequences preceding torsades de pointes or VF have been reported [44]. Accordingly, patients should be telemetry monitored during hospitalization until the documentation of QTc shortening, and β-blockers should be administered with caution [45]. 

In summary, patients diagnosed with TTS syndrome usually have a good outcome, characterized by resolution of AME, recovery of ventricular function and ECG abnormalities.

## 5. Resuscitated Sudden Cardiac Death

**Figure 3:**
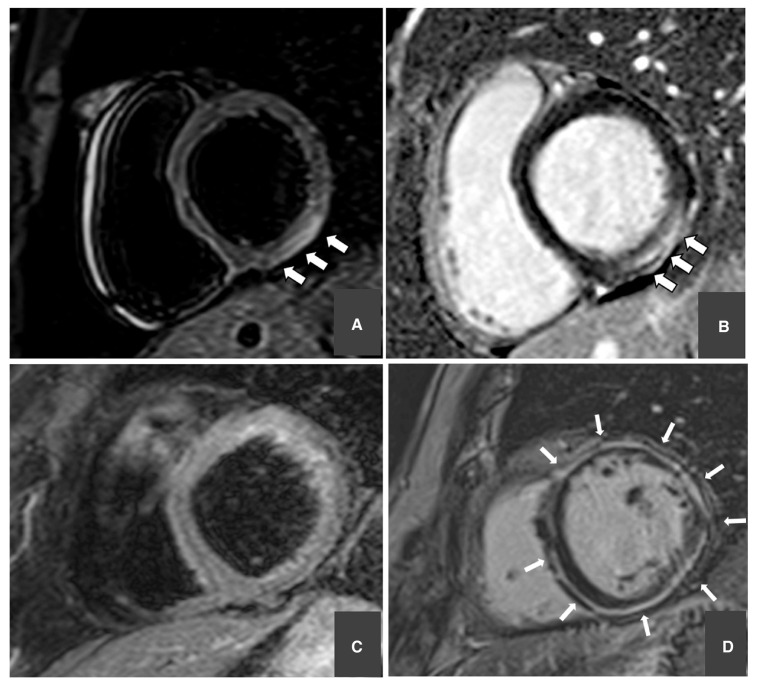
Cardiac magnetic resonance (CMR) T2-weighted (**left**) and late gadolinium enhancement (LGE) sequences (**right**). Short axis views showing subepicardial myocardial edema of the inferolateral left ventricular wall ((**A**) **arrows**) and subepicardial late gadolinium enhancement (LGE) in the same segment ((**B**) **arrows**) in a patient with normal coronary artery, suggesting acute myocarditis. Absence of myocardial edema (**C**) with circumferential subepicardial-midwall LGE ((**D**) **arrows**) in a patient with left-dominant arrhythmogenic cardiomyopathy. Adapted with kind permission from Zorzi et al. [34].

Recent studies pointed to the presence of AME in patients with resuscitated out of hospital cardiac arrest (OHCA) [46]. The pattern of distribution of edema varies upon the underlying conditions and has diagnostic implications. Indeed, a subendocardial to transmural extension of edema was found in patients with acute coronary syndrome related OHCA. On the other hand, midmyocardial or subepicardial pattern involving the infero-lateral LV wall was found in patients with diagnosis of acute myocarditis (Figure 3) and transmural pattern in the mid-apical LV segments in patients with diagnosis of TTS [47]. Aside its diagnostic value, myocardial edema holds prognostic value, in at least two ways. First, the detection of the exact cause of arrhythmias helps the cardiologist decide if implantable cardiac defibrillator (ICD) therapy is indicated. To date, there is a recommendation only in the evidence of a chronic or not reversible underlying disease [48]. Second, in the above-mentioned study, none of the patients with reversible myocardial edema reported episodes of arrhythmias in the next three years of follow-up, suggesting a favourable prognostic role of AME. These implications were further highlighted by Zorzi and colleagues in a study with broader population. In detail, among 101 patients who survived to OHCA, 89% of them with AME on baseline CMR had an uneventful long-term arrhythmic outcome. Furthermore, AME was associated with a lower risk of ICD therapy for major arrhythmias in the follow-up and emerged as an independent predictor of favourable outcome after adjustment for left ventricular ejection fraction and LGE [34]. In summary, myocardial edema demonstrated to hold both a diagnostic and a prognostic role in patients resuscitated from OHCA, suggesting a potentially great utility of CMR in their clinical management. 

## 6. Hypertrophic Cardiomyopathy

CMR is a cornerstone imaging technique in cardiomyopathies [49,50], and the presence of AME was investigated also in patients with hypertrophic cardiomyopathy (HCM). In a study by Melacini et al., edema was detectable in 24/44 (54%) HCM patients, more commonly in the most hypertrophied regions, also with concomitant patchy LGE and perfusion defects, thus suggesting an ischemic aetiology [51]. Indeed, a global reduction in myocardial blood flow has also been proven in these patients using positron emission tomography (PET) [52], and the extent of LGE has been shown to be inversely related to the global myocardial blood flow, thus suggesting a close relationship between microvascular disease, ischemic events and chronic myocardial damage [53]. The presence of edema in patients with HCM was shown to be a sign of advanced disease, progression and arrhythmogenesis. In a study by Todiere et al., edema was associated with lower ejection fraction, higher LV mass and greater LGE extent and arrhythmic burden [54]. The same results were recently proposed by Ziqian et al., who found that HCM patients with LGE and edema had a poorer outcome than patients with LGE but no edema [55]. 

## 7. Other Cardiac Diseases

AME was detected by CMR also in autoimmune diseases with possible cardiac involvement. Mavrogeni and colleagues demonstrated the presence of edema in over one-half of a cohort treatment of naïve patients with a broad spectrum of connective tissue diseases and without cardiovascular disease. Re-evaluation of the patients after 6 months of adequate disease-modifying treatment showed a significant reduction of AME [56]. Signs of myocardial edema were demonstrated in patients with systemic scleroderma [57], arthritis rheumatoid [58] and systemic lupus erythematosus [59]. Taken together, the available studies seem to indicate that myocardial edema could be an expression of subclinical inflammation in patient with autoimmune diseases. Therefore, an optimized disease-modifying treatment should be promptly provided to avoid further, irreversible injury and myocardial fibrosis replacement. A possible use of myocardial edema as an imaging marker for response to therapy is also suggested [56]. 

Similarly, myocardial edema was detected in cardiac amyloidosis (CA), both light chain (AL) and transthyretin (ATTR). In a study by Ridouani and colleagues of about 44 patients with CA, myocardial native T2 was significantly increased, especially in AL-CA [60]. Further evidence came from a study by Kotecha et al. in a study including 286 CA patients, both AL- and ATTR-CA. Myocardial T2 was overall increased, with the highest values in untreated AL patients. Treated patients achieved at least a very good partial response (52%), a partial response (16%) or no response (2%) to therapy. Presence of edema was proven in 87.5% of the 16 myocardial biopsies performed. Intriguingly, higher myocardial T2 resulted as an independent predictor for death in AL amyloidosis (but not in ATTR), also after adjustment for extracellular volume and N-terminal pro-B-type natriuretic peptide, suggesting that mechanisms other than amyloid infiltration could account for death in AL-CA [61]. Furthermore, these results highlight the importance of an adequate, possibly complete, response to chemotherapy. 

AME was also detected in heart transplant recipients with ongoing acute rejection confirmed by biopsy [62,63,64]. The possibility of non-invasive diagnosis of rejection by CMR could potentially avoid the current need of consecutive myocardial biopsies, with non-negligible risks for patients’ safety.

Myocardial edema was recognized in drug induced myocarditis as well, associated with immune checkpoint inhibitor (ICI) drugs and in the context of drug rash with eosinophilia and systemic symptoms (DRESS) syndrome [65,66]. AME was detectable in up to 43% of patients treated with ICI, and its prognostic role is not completely established. In a study by Thavendiranathan et al., the survival free from major adverse cardiovascular events was significantly lower in patients with abnormal T1 values, but no significant difference was seen with abnormal T2 values [67]. Furthermore, the presence of edema did not emerge as an independent predictor of outcome. These findings suggest the reversible nature of edema in this context and its reduction with an adequate therapy [68].

Eventually, AME was also detected in patients after COVID-19 infection, in absence of any clinical clues for acute myocarditis [69,70]. Its presence was not associated to any clinical or imaging evidence of poor prognosis.

## 8. Gaps of Evidence

There are some gaps of evidence that are worth addressing in future research. First, most of available studies assessed AME using T2-short tau inversion recovery imaging. Nevertheless, this technique is being replaced by T2 mapping, since its better reproducibility and spatial resolution. Indeed, few data are available about accuracy of AME detection with a combination of the two techniques. Second, the lack of standardized timing of CMR execution after acute injury could compromise the possibility of detection of AME, which is a transient phenomenon. This is particularly relevant in acute coronary syndromes, as previously described. Third, there is a scarcity of correlation studies between CMR detection of AME and histology. This could be not only of scientific interest but could also shed further light upon the prognostic significance of edema.

## 9. Future Perspectives

Multiple techniques are being considered to sort some relevant limitations in edema detection. As already reported, the pattern analysis is crucial to recognize the correct aetiology underlying AME. In this perspective, a layer-by-layer myocardial T2 analysis could be useful. Single-breath fat saturated dark-blood cardiac T2 mapping would improve accuracy of myocardial T2 mapping, by limiting partial-voluming related to both fat and blood, without significant loss of signal-to-noise ratio [71]. Other than diagnostic purposes, this technique could be also useful to elucidate the prognostic implications of myocardial edema. For example, in cardiac amyloidosis, a major amyloid deposition in trabecular and subendocardial layers has been documented [72]. A possible role of fibril-related inflammation could clarify the pathophysiology of numerous phenomena typically observed in CA, such apical sparing pattern of longitudinal strain on echocardiogram [72,73] and low QRS voltages on ECG [74,75].

Another technique that could improve the detection of AME with better sensitivity is diffusion-weighted imaging because of its contrast mechanism [76]. This has already been tested in acute and chronic coronary syndromes [77,78,79,80]. Nevertheless, its most intriguing usefulness relies upon the enabled analysis of the myocardial fibres architecture and, consequently, the microstructural changes in numerous myocardial pathologic conditions, AME included [81].

## 10. Conclusions

**Figure 4:**
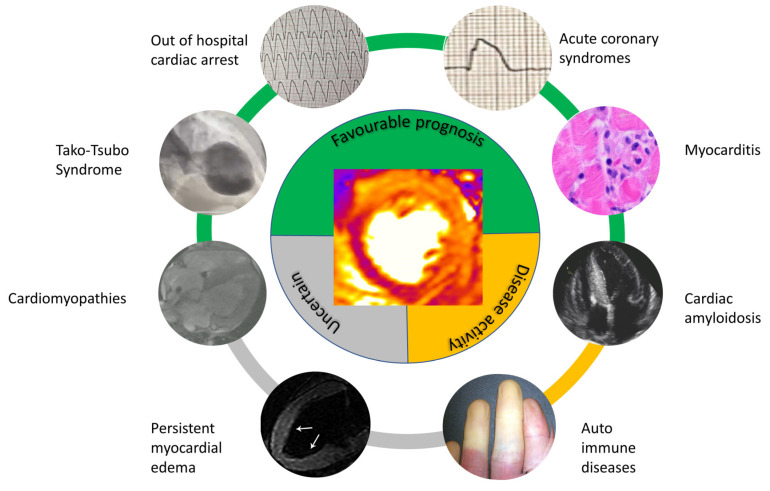
Central Figure, Prognostic significance of myocardial edema in different clinical scenarios.

Acute myocardial edema is a hallmark of myocardial injury, whatever the underlying etiology. The progress in cardiovascular magnetic resonance techniques, particularly with T2 weighted sequences and T2 mapping, allows its detection in multiple clinical scenarios. Generally, its presence owns a favourable prognostic meaning in widespread common cardiac conditions, such as acute coronary syndromes, acute myocarditis, TTS and OHCA (Table 1) (Figure 4. Central Figure). In this regard, the aphorism has been proposed as “myocardial edema: bonum et laudabile” to highlight the evolving perspective that the visualization of AME by CMR reflects an acute and potentially reversible tissue change and thus may portend a benign prognosis [1].

By contrast, in the context of systemic disorder as autoimmune diseases or cardiac amyloidosis, its presence is a signal of none or non-optimal treatment. Further studies are needed to overcome current limitations in the assessment of AME and to elucidate the prognostic meaning in less explored fields, such as persistent edema, autoimmune disease, cardiomyopathies and heart transplantation.

**Table 1:** Prevalence and clinical significance of myocardial edema in different cardiac disease.

Disease	Study	*n*	Time of Scanning	Prevalence	Prognostic Index	Outcome
**Acute coronary syndromes**	[12]	208	1–4 days	100%	MSI extension	Less MACE at 6 months
	[13]	92	3–5 days	100%	MSI extension	Less re–AMI at 263 days
**Acute myocarditis**	[26]	388	2–6 days	63%	LGE without edema	More MACE at 15 days
	[28]	187	1–7 days 6 months	96% 16%	LGE without edema	More MACE at 7 years
**TakoTsubo syndrome**	[39]	199	2–4 days 6 months	81% 0%	LVEF and isolated AME	Normalization of LVEF and AME resolution at 6 months
	[40]	20	3 days 3 months	100% 0%	LVEF and isolated AME	Normalization of LVEF and AME resolution at 3 months
**OHCA**	[47]	44	1–7 days	41%	Isolated AME	No arrhythmic event at 3 years
	[34]	101	8–22 days	18%	Isolated AME	No appropriate ICD therapy at 47 months
**HCM**	[54]	65	Time of diagnosis	42%	Isolated AME	Higher risk of LV arrhythmias
	[55]	674	Time of diagnosis	42%	LGE with edema	More risk of CVE at 36 months.
**CA**	[61]	286	Time of diagnosis	100% AL 0% ATTR	Isolated AME	More risk of death at 23 months
**Autoimmune disease**	[56]	78	Time of diagnosis	56%	Isolated AME	Reduction of AME after appropriate therapy

Abbreviations: AL = light chain amyloidosis; ATTR = transthyretin amyloidosis; AME = acute myocardial edema; AMI = acute myocardial infarction; CA = cardiac amyloidosis; CVE = cardiovascular events; HCM = hypertrophic cardiomyopathy; ICD = implantable cardioverter defibrillator; LGE = late gadolinium enhancement; LV = left ventricle; LVEF = left ventricle ejection fraction; MACE = major adverse cardiovascular events; MSI = myocardial salvage index; OHCA = out of hospital cardiac arrest.

## Data Availability

Not applicable.
